# Viscosity, Morphology, and Thermomechanical Performance of Attapulgite-Reinforced Bio-Based Polyurethane Asphalt Composites

**DOI:** 10.3390/polym17152045

**Published:** 2025-07-26

**Authors:** Haocheng Yang, Suzhou Cao, Xinpeng Cui, Zhonghua Xi, Jun Cai, Zuanru Yuan, Junsheng Zhang, Hongfeng Xie

**Affiliations:** 1 MOE Key Laboratory of High Performance Polymer Materials and Technology, School of Chemistry and Chemical Engineering, Nanjing University, Nanjing 210023, China; 522023240043@smail.nju.edu.cn (H.Y.); 522024240001@smail.nju.edu.cn (S.C.); 15299908527@163.com (X.C.); 2 Experimental Chemistry Teaching Center, School of Chemistry and Chemical Engineering, Nanjing University, Nanjing 210023, China; xizh@nju.edu.cn; 3 Public Instrument Center, School of Chemistry and Chemical Engineering, Nanjing University, Nanjing 210023, China; caijun@nju.edu.cn; 4 Modern Analysis Center, Nanjing University, Nanjing 210023, China; zryuan@nju.edu.cn

**Keywords:** polyurethane asphalt, bio-based polyurethane, phase separation, cure kinetics, glass transition temperature

## Abstract

Bio-based polyurethane asphalt binder (PUAB) derived from castor oil (CO) is environmentally friendly and exhibits extended allowable construction time. However, CO imparts inherently poor mechanical performance to bio-based PUAB. To address this limitation, attapulgite (ATT) with fibrous nanostructures was incorporated. The effects of ATT on bio-based PUAB were systematically investigated, including cure kinetics, rotational viscosity (RV) evolution, phase-separation microstructures, dynamic mechanical properties, thermal stability, and mechanical performance. Experimental characterization employed Fourier transform infrared spectroscopy, Brookfield viscometry, laser scanning confocal microscopy, dynamic mechanical analysis, thermogravimetry, and tensile testing. ATT incorporation accelerated the polyaddition reaction conversion between isocyanate groups in polyurethane (PU) and hydroxyl groups in ATT. Paradoxically, it reduced RV during curing, prolonging allowable construction time proportionally with clay content. Additionally, ATT’s compatibilizing effect decreased bitumen particle size in PUAB, with scaling proportionally with clay loading. While enhancing thermal stability, ATT lowered the glass transition temperature and damping properties. Crucially, 1 wt% ATT increased tensile strength by 71% and toughness by 62%, while maintaining high elongation at break (>400%). The cost-effectiveness and significant reinforcement capability of ATT make it a promising candidate for producing high-performance bio-based PUAB composites.

## 1. Introduction

Polyurethane (PU), a highly versatile polymer, has become ubiquitous in modern applications. Global PU production reached approximately 22 million tons in 2023, representing 5.3% of total polymer output and ranking fifth among all polymers produced, trailing polyethylene, polypropylene, polyvinyl chloride, and polyethylene terephthalate [1]. Due to the versatility offered by their chemical composition and tunable molecular architectures, PU materials have found extensive applications across multiple industries. Key implementations include thermal insulation foams, protective coatings, structural adhesives, engineering elastomers, and industrial sealants [2,3,4].

Recent years have witnessed growing research attention toward polyurethane as a thermosetting asphalt modifier [5,6,7]. In contrast to conventional thermoplastic-polymer-modified asphalts (e.g., styrene–butadiene–styrene, SBS), polyurethane asphalt (PUA), also known as polyurethane-modified asphalt, demonstrates superior performance grade (PG) elevation, enhanced compatibility, deformation resistance, and water resistance, albeit with comparatively reduced low-temperature performance [8,9]. Notably, akin to established thermosetting-polymer-modified asphalt systems (e.g., epoxy asphalt) [10], PUAs featuring continuous three-dimensional crosslinked networks demonstrate intrinsic insolubility and non-thermoplastic behavior. This structural transformation fundamentally restricts the mobility of bitumen fractions while transitioning the composite from thermoplastic to thermosetting properties [11]. Such engineered viscoelastic properties position PUAs as advanced binders for critical infrastructure applications, particularly in steel bridge deck pavements requiring exceptional load-distribution capacities [12].

Plastics Europe data [1] reveal that over 99% of commercial polymers remain petrochemically sourced, with bio-based variants constituting merely 0.7% of global production in 2023. This stark reliance on finite fossil resources, coupled with escalating ecological imperatives, has catalyzed intensive research into renewable alternative thermosetting polymers [13,14]. The polyurethane sector exemplifies this transition, where biorefinery-derived polyols, sourced from lipidic (plant oil and fatty acids) and lignocellulosic (crop residues and lignin) feedstocks, now enable large-scale biosynthesis [15,16,17]. Significantly, these sustainable PU systems are increasingly developed as asphalt modifiers, demonstrating circular economy potential in infrastructure materials [18,19,20].

Due to its naturally occurring hydroxyl groups, cost-effectiveness, and commercial availability, castor oil (CO)-modified bitumen and PUA production have garnered increasing attention. Xia et al. [21] demonstrated that incorporating CO-based PU prepolymers into bitumen increases the softening point, ductility, and complex modulus while decreasing the phase angle of bitumen. Cuadri et al. [22] utilized 2 wt% CO-based PU prepolymer to modify bitumen, enhancing its resistance to permanent deformation due to the chemical reactions between −NCO groups and air moisture, as well as bitumen components. Kazemi et al. [23] showed that adding 9 wt% CO-based PU increases the tensile strength ratio and bond strength under both dry and wet conditions by 49%, 55.73%, and 37.93%, respectively. Notably, most CO-based PUAs prepared by PU prepolymers or at low PU loading levels exhibit poor performance because of their bitumen-dominated phase. In contrast to petroleum-based polyols, CO-based PU inherently possesses poor mechanical properties (e.g., low tensile strength and toughness) due to its high crosslinking density and flexible backbone [24]. Consequently, CO-based PUA with a PU-dominated phase also displays inadequate mechanical performance, limiting its use in advanced infrastructure applications (e.g., steel bridge deck). Our previous study [25] systematically examined the correlation between the isocyanate index (R-value, defined as [NCO]/[OH] molar ratio) and the performance of CO-based polyurethane asphalt binder (PUAB). The formulation with R = 1:1.2 demonstrates optimal processing characteristics: extended allowable construction time (time to reach 1 Pa·s > 60 min at 120 °C) and superior damping capacity (maximum damping factor, (tan δ)_max_ > 1.0 and effective damping range of tan δ > 0.3, ΔT > 55 °C). However, it exhibits insufficient tensile strength, failing to satisfy GB/T 30598 requirements for thermosetting-polymer-modified asphalt (>1.50 MPa) [26]. To mitigate the high crosslinking density and enhance ductility, rigid-structured compounds have been incorporated into the backbone of CO-based polyurethane [27]. Meng et al. [28] introduced renewable and stiff isosorbide, which contains a secondary hydroxyl group, to improve the mechanical performance of CO-based PUA. The resulting bio-based PUA exhibits good compatibility, suitable workability, and satisfactory performance.

Attapulgite (ATT), a naturally occurring magnesium aluminosilicate clay mineral (chemical formula: (Mg, Al)_2_Si_4_O_10_(OH)·4H_2_O), finds widespread applications across agricultural, chemical, and environmental sectors due to its exceptional adsorption capacity and colloidal properties [29]. Characterized by its unique one-dimensional nanostructure, ATT features needle-like crystals (20–70 nm in diameter and 0.5–5 µm in length) with surface hydroxyl groups (−OH) that enable covalent bonding with polymer matrices [30]. Beyond its well-documented reinforcement effects in polyurethane composites [31,32], ATT demonstrates multifunctional benefits in asphalt modification, including (1) VOC (volatile organic compound) reduction: 81.5% decrease in total emissions with 8 wt% ATT/diatomite (1:1) hybrid filler [33], (2) odor suppression: 71.6% reduction in crumb rubber modified asphalt emissions at 5 wt% loading [34], (3) aging resistance: 70% and 67% lower carbonyl index of styrene–butadiene–styrene (SBS)-modified asphalt after thermal and UV aging tests [35], (4) self-healing enhancement: 82.67% release of rejuvenator. Recent advances show ATT’s effectiveness in improving the aging and low- and high-temperature performance of PUAs [36]. Nevertheless, its application in CO-based polyurethane asphalt remains underexplored, particularly regarding phase compatibility and the bio-composite interfacial bonding mechanism.

**Figure 1:**
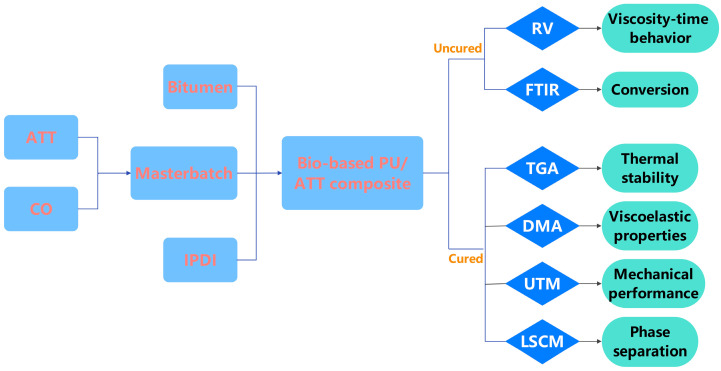
Flow chart of bio-based PUAB/ATT composite preparation and characterization.

In this study, fibrous ATT was used to reinforce CO-based PUAB. To achieve this goal, ATT was incorporated into CO to produce a masterbatch. Subsequently, the masterbatch was mixed with isophorone diisocyanate (IPDI) and bitumen to prepare bio-based PUAB. The cure kinetics and time-dependent rotational viscosity (RV) characteristics of the uncured PUAB/ATT composite were characterized by attenuated total reflectance Fourier-transform infrared (ATR-FTIR) spectroscopy and a Brookfield rotational viscometer. The mechanical performance, thermal stability, viscoelastic properties, and phase-separated microstructures of cured bio-based PUAB/ATT composites were investigated by a universal testing machine (UTM), thermogravimetric analysis (TGA), dynamic mechanical analysis (DMA), and laser scanning confocal microscopy (LSCM). Figure 1 illustrates the flow chart of the preparation and characterization of bio-based PUAB/ATT composites.

## 2. Materials and Methods

### 2.1. Materials

Bitumen of a 60/80 penetration grade was sourced from China Offshore Bitumen (Taizhou) Co., Ltd. (Taizhou, China), with its key physicochemical characteristics detailed in Table 1. CO with a molecular weight of 928 g/mol and functionality of 2.7 was procured from Bide Pharmtech Co., Ltd. (Shanghai, China). IPDI (industrial grade) was obtained from Yantai Wanhua Polyurethanes Co., Ltd. (Yantai, China). Pristine ATT was provided by Jiangsu Golden Stone Attapulgite R&D Co., Ltd. (Huaian, China). Table 2 summarizes the chemical composition of ATT determined by X-ray fluorescence spectroscopy (XRF).

### 2.2. Preparation of Bio-Based PUAB/ATT Composites

**Figure 2:**
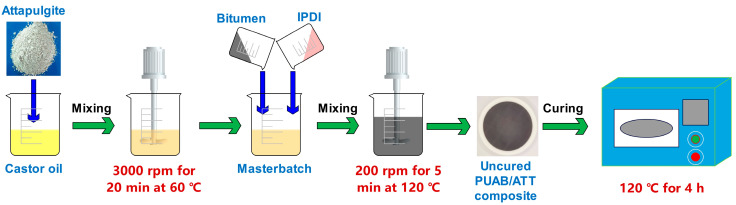
Schematic of the bio-based PUAB/ATT composite fabrication process.

The fabrication protocol for bio-based PUAB/ATT composites comprised three sequential steps (Figure 2):

(1) Masterbatch preparation: CO and ATT were homogenized using an FM300 high-shear mixer (Fluko, Shanghai, China) (50 s^−1^, 60 °C, 20 min).
(2) Reactive blending: Preheated bitumen (120 °C, 30 min) and IPDI were sequentially introduced into the masterbatch. The mixture was stirred under controlled conditions (3.3 s^−1^, 120 °C, 5 min).
(3) Curing: The reactive composite was cast into a polytetrafluoroethylene (PTFE) mold (Φ 100 mm × 5 mm) and thermally cured (120 °C, 4 h) to achieve full crosslinking.

Based on our previous studies [25,43], the molar ratio of IPDI to CO was maintained at 1:1.2. The bitumen content in bio-based PUAB was 60 wt%. The ATT content in the composites was varied at 0.5, 1, and 2 wt%.

### 2.3. Methods

#### 2.3.1. RV

Rotational viscosity was measured using an NDJ-1 Brookfield viscometer (Shanghai Changji Instrument Co., Ltd., Shanghai, China) as per ASTM D4402-06 [40]. Measurements employed spindle #18 at 120 °C and 0.8 s^−1^ shear rate, with continuous monitoring until reaching the critical viscosity of 5 Pa·s.

#### 2.3.2. FTIR Spectroscopy

FTIR spectra were acquired using an FTIR spectrometer (Alpha, Bruker, Ettlingen, Germany) equipped with a single-reflection diamond ATR module. Data collection covered the 400−4000 cm^−1^ range at 8 cm^−1^ and 16 co-added scans. Curing monitoring was performed at 120 °C with 30 min intervals throughout the 240 min cure cycle.

#### 2.3.3. DMA

The dynamic mechanical properties were measured in a tension mode using a 01 dB-Metravib DMA + 450 analyzer (Limonest, France). The samples (25 mm × 15 mm × 2.5 mm) were tested at 1 Hz oscillation frequency and 0.1% strain amplitude with a heating rate of 3 °C/min over −50 to 100 °C.

#### 2.3.4. DSC

Differential scanning calorimetry was conducted using a Perkin-Elmer Pyris 1 instrument (Norwalk, CT, USA) with continuous argon purging (20 mL/min). Approximately 5 mg samples in sealed aluminum crucibles underwent five thermal treatment stages (Table 3).

#### 2.3.5. TGA

Thermal stability was evaluated using a Mettler Toledo TGA/DSC1 instrument (Zurich, Switzerland) under a continuous nitrogen atmosphere (20 mL/min). Samples (~10 mg) in alumina crucibles were heated at 20 °C/min from 50 °C to 600 °C.

#### 2.3.6. LSCM

Phase-separated microstructures were observed using a Zeiss LSM710 confocal microscope (Jena, Germany) equipped with an argon-ion excitation source (488 nm). The samples were prepared as follows: The uncured bio-based PUAB/ATT mixture was deposited onto a preheated glass slide on a 120 °C hot stage. A coverslip was placed over the droplet and gently pressed to form a uniform film. The assembly was thermally cured (120 °C, 4 h).

Image-Pro Plus 6.0 was used to analyze the particle size distributions and average diameters, calculated by Equations (1) and (2):

(1) $$D_{n}=\frac{\Sigma n_{i}D_{i}}{\Sigma n_{i}}$$

(2) $$D_{w}=\frac{\Sigma n_{i}{D_{i}}^{2}}{\Sigma n_{i}D_{i}}$$

where *n*_i_ denotes the count of domains with diameter *D*_i_.

#### 2.3.7. Uniaxial Tensile Testing

Mechanical performance was evaluated using an AGX-10kNVD UTM (Shimadzu, Kyoto, Japan) equipped with a 10 kN load cell. Following ASTM D638 Type V specifications [44], five dog-bone-shaped specimens were measured at a crosshead speed of 200 mm/min. The average values with their standard deviations were presented to ensure experimental repeatability.

## 3. Results and Discussion

### 3.1. Structural Characterization

**Figure 3:**
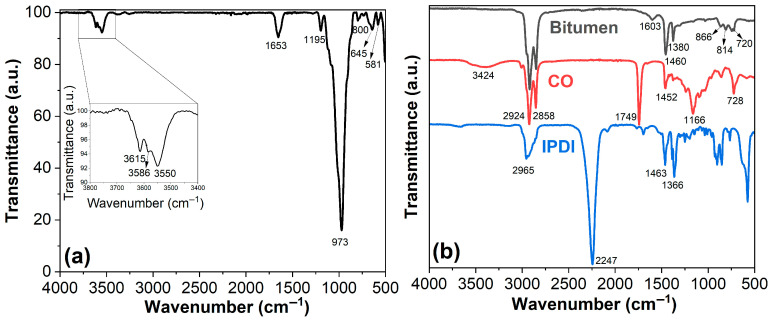
FTIR spectra of ATT (**a**), bitumen, CO, and IPDI (**b**).

Figure 3 demonstrates the FTIR spectra of the raw materials for bio-based PUAB/ATT composites; corresponding characteristic peak assignments are summarized in Table 4. In the high wavenumber region (3800−3400 cm^−1^) of the ATT spectrum (Figure 3a), the peaks at 3615, 3586, and 3550 cm^−1^ are attributed to −OH stretching vibrations of Al–Al–OH, Al-Fe-OH, and bound water, respectively [45]. In the low wavenumber range (1700−500 cm^−1^), the 1653 cm^−1^ band corresponds to the bending vibration of bond water [46]. The 1195 cm^−1^ band is associated with Si–O–Si stretching vibration. The sharp peak at 973 cm^−1^ is associated with metal−OH (M–OH) deformation [47]. Peaks at 800, 645, and 581 cm^−1^ are assigned to correspond to Si–O–Si symmetric stretching vibration (quartz), H_2_O–Mg–H_2_O stretching vibrations, and AlO_6_ octahedron vibrations, respectively [48].

As shown in Figure 3b, peaks at 1603, 866, and 814 cm^−1^ in the bitumen spectrum correspond to aromatic ring vibrations. Additional peaks at 1460 cm^−1^ (–CH_2_– scissoring vibration), 1380 cm^−1^ (–CH_3_ umbrella vibration), and 720 cm^−1^ (rocking vibration of –(CH_2_)_n_–, where *n* ≥ 4) indicate aliphatic structures [49].

The broad peak near 3424 cm^−1^ in the CO spectrum is assigned to –OH stretching vibrations. Peaks at 2924 cm^−1^ (asymmetric) and 2858 cm^−1^ (symmetric) are characteristic of C–H stretching in –CH_2_ groups, whereas peaks at 1749 cm^−1^ (C=O stretching) and 1166 cm^−1^ (C–O–C stretching) confirm ester functionalities [50].

The sharp peak at 2247 cm^−1^ in the IPDI spectrum indicates –NCO groups, while bands at 1463 cm^−1^ (–CH– deformation) and 1366 cm^−1^ (–CH_2_ bending) further validate aliphatic structures [51].

**Figure 4:**
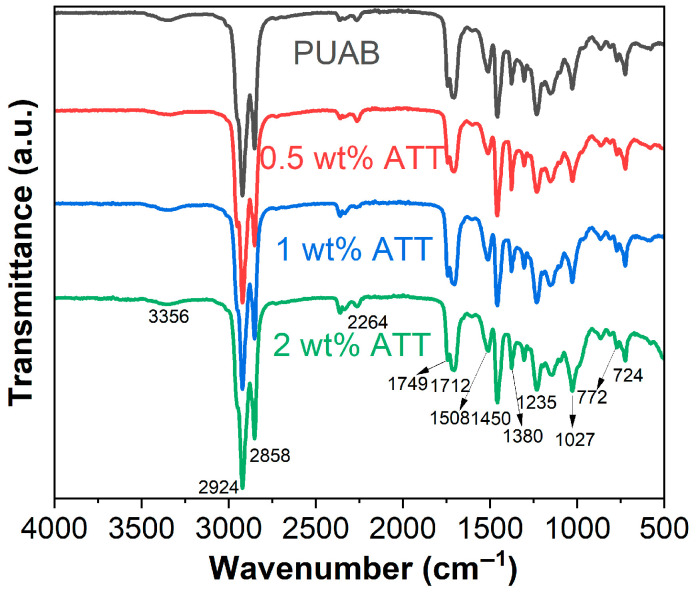
FTIR spectra of cured bio-based PUAB and PUAB/ATT composites.

Figure 4 presents the FTIR spectra of cured PUAB and its ATT composites. The significant decrease in intensity of the –NCO peak at 2264 cm^−1^ in PUAB indicates the reaction between –NCO groups (IPDI) and −OH groups (CO), forming urethane linkages (–NH–CO–O). Characteristic absorption bands at 3356, 1712, 1511, and 772 cm^−1^ are attributed to –NH stretching, amide I: C=O stretching, N–H in-plane bending, and N–H out-of-plane bending, respectively. These confirm polyurethane network formation [52]. In PUAB/ATT composites, hydroxyl-related peaks (3615, 3586, 3550, 1653, and 973 cm^−1^) disappear due to reactions with IPDI’s –NCO groups.

### 3.2. Cure Behavior

**Figure 5:**
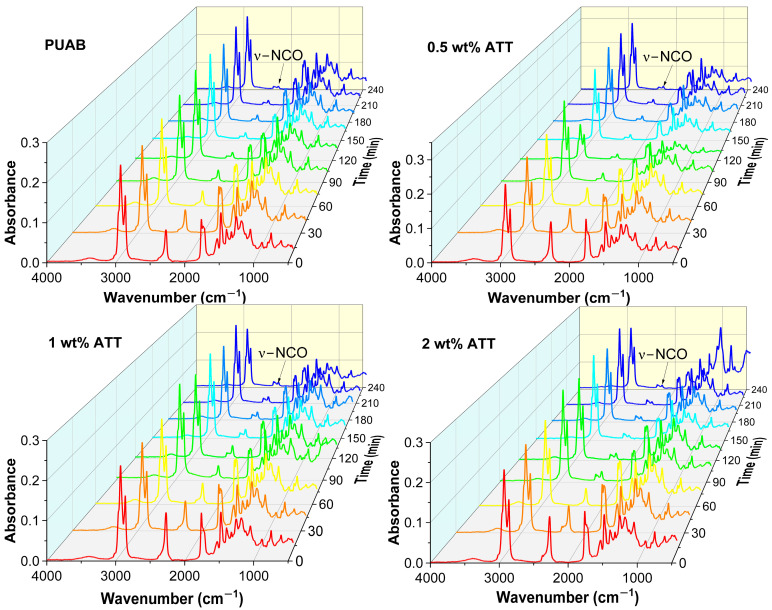
FTIR spectra of bio-based PUAB and PUAB/ATT composites during 120 °C curing.

Figure 5 displays FTIR spectra tracking curing progression for bio-based PUAB and its ATT composites. The continuous decrease in intensity of the –NCO stretching band at 2264 cm^−1^ confirms ongoing polyaddition between isocyanates and polyols [53]. After 4 h of curing, a low-intensity –NCO band remains detectable, indicating incomplete reaction.

To quantitatively evaluate ATT’s effect on the cure kinetics of bio-based PUAB, conversion was calculated using Equation (3):

(3) $$\alpha =\left(1-\frac{A_{NCO,t}/A_{R,t}}{A_{NCO,0}/A_{R,0}}\right)\times 100\%$$

where

- *A*_NCO,0_ and *A*_NCO,t_ = absorbance of the –NCO peak (2264 cm^−1^) at initial time (*t*_0_) and time *t*;
- *A*_R,0_ and *A*_R,t_ = absorbance of the internal reference peak (2924 cm^−1^) at corresponding times.

**Figure 6:**
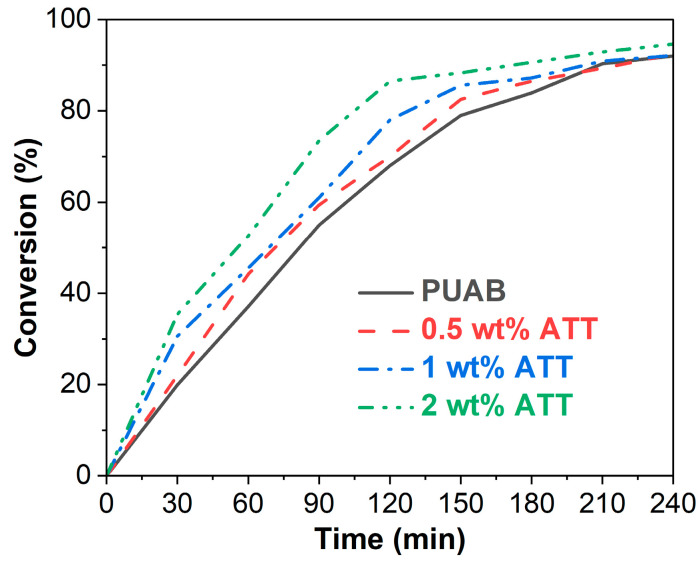
Conversion as a function of time for bio-based PUAB and PUAB/ATT composites at 120 °C.

Figure 6 illustrates the conversion of bio-based PUAB and its ATT composites during curing at 120 °C. Prior to 210 min, ATT composites exhibit higher conversions than bio-based PUAB, suggesting a catalytic acceleration of the isocyanate–polyol polyaddition by hydroxyl groups of attapulgite. Beyond 210 min, ATT’s influence becomes negligible except at 2 wt% loading. Conversion consistently increases with ATT loading across all composites. As summarized in Table 5, after 4 h curing at 120 °C, bio-based PUAB and its ATT composite (<1 wt%) achieve ~92.0% conversion, while the 2 wt% ATT composite reaches 94.6%. These values indicate insufficient conversion due to either kinetic limitations or reactant depletion.

### 3.3. Time-Dependent Rotational Viscosity Behavior

**Figure 7:**
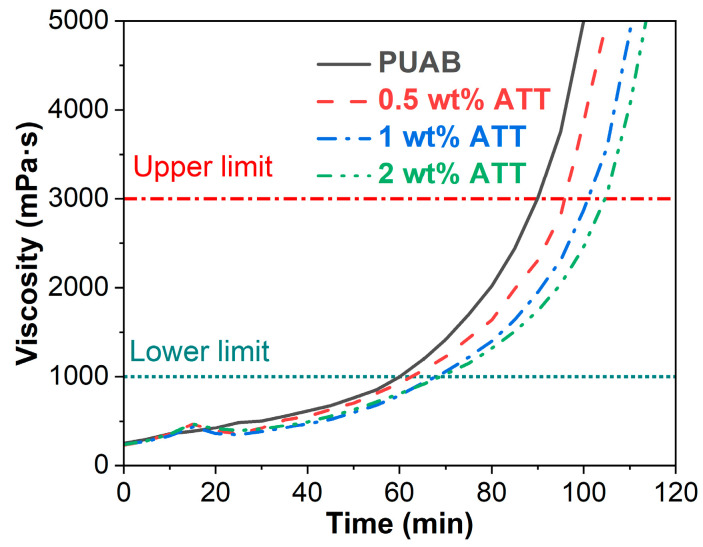
Rotational viscosity over time for bio-based PUAB and PUAB/ATT composites at 120 °C.

Similar to other thermosetting-polymer-modified asphalts, bio-based PUAB exhibits both temperature- and time-dependent rotational viscosity due to reactions between −NCO groups in IPDI and −OH groups in CO [25]. Consequently, the reactivity of the PU system must be precisely controlled to prevent excessive viscosity buildup that would shorten the paving window and compromise compaction quality. Figure 7 illustrates the rotational viscosity versus curing time for bio-based PUAB and its ATT composites at 120 °C. The rotational viscosity of neat PUAB progressively increases with curing time, attributed to the polyaddition reaction between isocyanate and polyol components. Within the initial 10 min period, ATT exhibits minimal influence on the rotational viscosity of neat PUAB. However, between 10 and 20 min curing, the ATT composites display higher viscosity values compared to neat PUAB, resulting from the catalytic activity of hydroxyl groups present in the attapulgite structure as discussed in Section 3.1 (Figure 3a). Notably, the relationship between ATT content and composite viscosity is nonlinear. After 20 min of curing, the composites demonstrate lower viscosity than neat PUAB, with the viscosity value decreasing proportionally with increasing ATT loading. The retarding effect of ATT is attributed to its thixotropic and non-Newtonian behavior [29]. A similar influence has been observed in warm-mix epoxy asphalt binder (WEAB) and bond coat composites [43,54].

In contrast to its catalytic effect on the polyaddition reaction (Figure 6), ATT incorporation delays the viscosity increase of neat PUAB (Figure 7), indicating competitive dynamics between catalytic and retarding effects during curing. Crucially, the high bitumen content (60 wt%) in PUAB substantially dilutes the polyurethane polyaddition reaction, enabling the retarding effect to dominate over catalysis.

According to the Strategic Highway Research Program (SHRP) [55], the maximum viscosity of bitumen should not exceed 3 Pa·s. For WEAB, the time to reach viscosities of 1 and 3 Pa·s defines the lower limit of reserved time (prior to paving and compaction) and the upper limit of allowable construction time (during paving and compaction) [56]. As shown in Table 6, ATT composites exhibit longer times to reach these viscosities compared to neat PUAB, indicating extended allowable construction time due to the incorporation of attapulgite. Additionally, the allowable construction time of PUAB/ATT composites progressively increases with higher ATT content. With the addition of 0.5−2 wt% ATT, the time for bio-based PUAB to reach 1 Pa·s rises from 60 min to 63, 68, and 69 min and to reach 3 Pa·s from 90 min to 96, 101, and 105 min.

### 3.4. Phase-Separated Morphology

**Figure 8:**
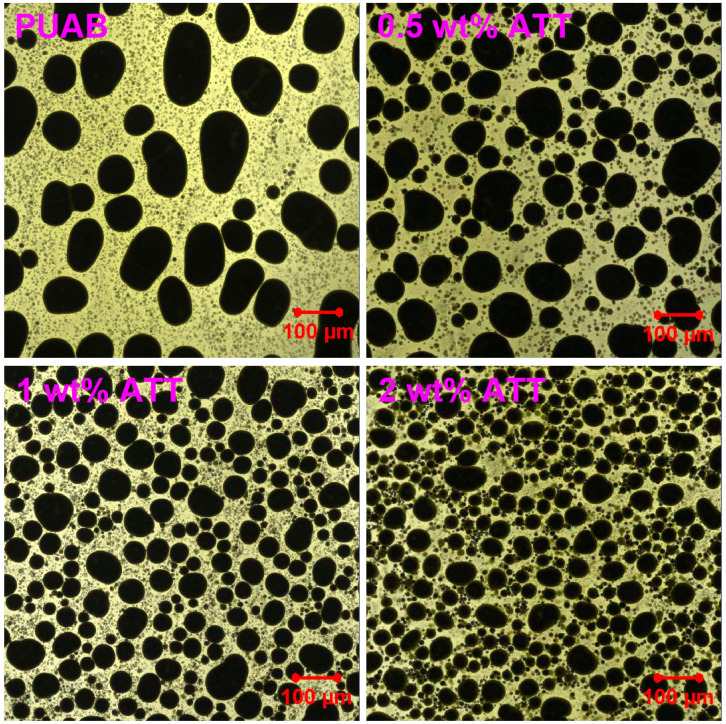
LSCM micrographs of bio-based PUAB and PUAB/ATT composites.

Figure 8 demonstrates LSCM micrographs of cured bio-based PUAB and its ATT composites. Because of the immiscibility between bitumen and crosslinked thermosetting polymers, phase separation inevitably occurs in thermosetting-polymer-modified asphalt [57]. Moreover, the resulting morphology is determined by the bitumen-to-thermosetting polymer mass ratio [58]. Despite the high bitumen content (60 wt% or 61.7 vol.%), bio-based PU forms the continuous phase (yellow), while irregularly shaped spherical bitumen particles (black) are dispersed in the continuous PU phase. The incorporation significantly reduces the size of bitumen particles dispersed in the continuous PU phase.

**Figure 9:**
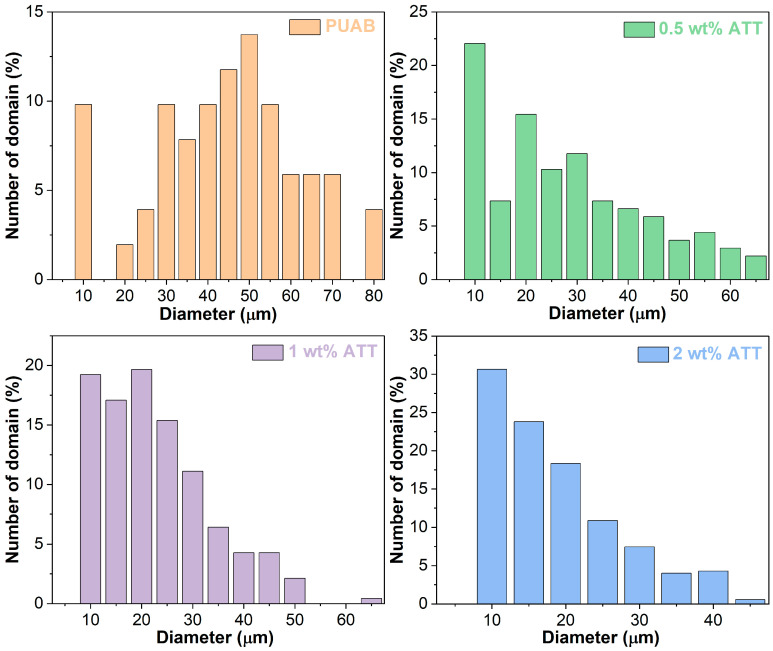
Particle size distribution of bitumen domains in bio-based PUAB and PUAB/ATT composites.

Figure 9 presents particle size distributions of bitumen particles in bio-based PUAB and its ATT composites. The size of bitumen particles in neat PUAB is primarily distributed between 20 and 70 µm. In the ATT composites, the distribution shifts significantly toward smaller sizes, specifically ranging from 10 to 60 µm, 10 to 50 µm, and 10 to 40 µm with increasing clay loading.

Table 7 summarizes the average diameters and dispersity (Ɖ, defined as the ratio of *D*_w_ to *D*_n_) of bitumen particles in cured bio-based PUAB and its ATT composites. The results demonstrate that ATT incorporation significantly reduces the average particle diameters in bio-based PUAB. Furthermore, the average diameter of bitumen particles in ATT composites shows a decreasing trend with increasing clay loading. Specifically, with 2 wt% ATT addition, the *D*_n_ and *D*_w_ values decrease from 41.4 to 15.8 µm and from 50.7 to 21.6 µm, respectively. ATT incorporation also increases the dispersity of bio-based PUAB, suggesting a more heterogeneous distribution of bitumen particles in the composites compared to neat PUAB. Notably, the dispersity of ATT composites displays a nonlinear dependence on clay content.

The reduction in bitumen particle size is attributed to the compatibilizing effect of the fibrous clay [59]. The phase separation of polymer blends is due to high interfacial tension between components. Additionally, phase-separated particles may tend to coalesce, resulting in large particle sizes for dispersed domains [60]. The high interfacial tension between polymer components in blends can be reduced by adding interfacial agents, known as compatibilizers. These molecules localize at the interface between phases, reducing interfacial tension and thereby enhancing blend compatibility. Uncured PUAB forms a homogeneous mixture due to the favorable compatibility between castor oil and bitumen. However, chemical reactions of isocyanate (from IPDI) and hydroxyl groups (from CO) generate crosslinking networks within PUAB. This progressively disrupts the castor oil and bitumen compatibility and amplifies interfacial tension between polyurethane and bitumen, ultimately triggering phase separation in the system. In PUAB composites, ATT plays three primary roles in the phase separation process of bio-based PUAB [61]: (1) reducing interfacial tension, which promotes droplet breakup during curing; (2) inhibiting droplet coalescence, thereby stabilizing the morphology of the polymer blend; (3) reacting with compounds containing carboxylic acid in bitumen, enhancing interfacial bonding while restricting droplet growth.

### 3.5. Dynamic Mechanical Properties

**Figure 10:**
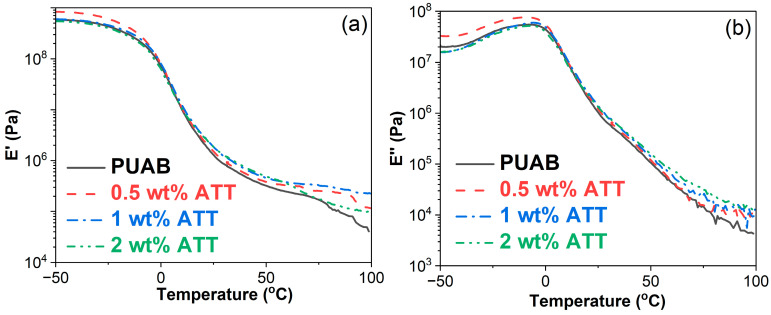
Storage modulus (**a**) and loss modulus versus temperature (**b)** of bio-based PUAB and PUAB/ATT composites.

Figure 10 illustrates storage modulus (E′) and loss modulus (E″) as functions of temperature for bio-based PUAB and its ATT composites. The influence of ATT on the dynamic modulus of bio-based PUAB can be divided into three distinct regions: (1) −50−0 °C, where the 0.5 wt% ATT composite exhibits higher E′ and E″ than neat PUAB, while other composites show nearly identical E′ and E″ values to neat PUAB; (2) 0−15 °C, where both bio-based PUAB and its ATT composites demonstrate comparable E′ and E″ values; (3) 15−100 °C, where all ATT composites display superior E′ and E″ values compared to neat PUAB.

**Figure 11:**
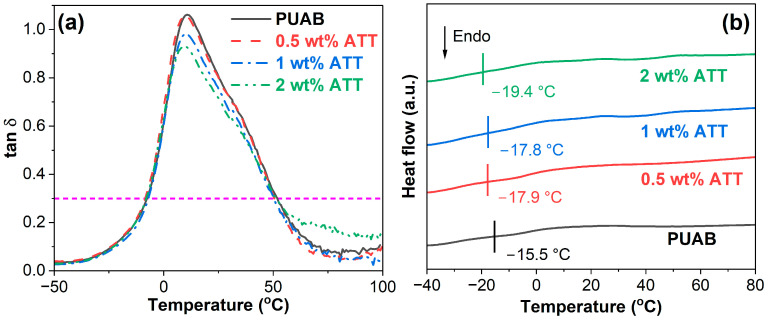
Damping factor–temperature (**a**) and DSC curves (**b**) of bio-based PUAB and PUAB/ATT composites.

The *T*_g_s of neat PUAB and its ATT composites were determined from the peak maxima of the E″ and tan δ curves in Figure 10b and Figure 11a. As shown in Table 8, the incorporation of ATT slightly lowers the *T*_g_ of neat PUAB. However, the *T*_g_ of ATT composites shows a nonlinear relationship with clay loading. Similar trends are also observed in the tan δ–temperature and DSC curves (Figure 11). Sun et al. [43] reported that ATT incorporation slightly reduces the *T*_g_ of WEAB. Notably, due to crosslinking networks, the DSC-measured *T*_g_ values of PUAB and its ATT composites are significantly higher than the *T*_g_ of neat bitumen (−26.0 °C) [58].

It is well established that the *T*_g_ of polymers is dependent on crosslinking density (ν_e_), calculated via Equation (4):

(4) $$\nu_{e}=\frac{{E^{\prime }}_{T}}{3RT}$$

where *R* = gas constant. *T* = rubbery-state temperature at (*T*_g_ + 40 K). *E’*_T_ = rubbery-state storage. As Table 8 demonstrates, 2 wt% ATT loading reduces ν_e_ in neat PUAB, whereas the addition of 0.5 wt% and 1 wt% ATT increases it. Additionally, the ν_e_ in ATT-modified PUAB decreases progressively with higher ATT loading. This observed *T*_g_ reduction could be attributed to two main factors: (1) the presence of moisture and excessive hydroxyl/organic groups in pristine ATT, which hinders effective interfacial interaction between polyurethane and attapulgite [62], and (2) the plasticizing effect of moisture, which simultaneously decreases both the *T*_g_ and mechanical strength of polyurethane [63]. Notably, this reduction in *T*_g_ enhances the low-temperature performance of bio-based PUAB [64]. However, thermal treatment and purification of ATT enhance the *T*_g_ of the polyurethane matrix [65,66].

### 3.6. Damping Properties

As depicted in Table 9, neat PUAB demonstrates a maximum tan δ ((tan δ)_max_) of 1.06. The effective damping range (Δ*T*), defined as the interval where tan δ > 0.3, reaches 59.8 °C, while the integrated area under the tan δ−temperature curve (*TA)* measures 41.8 K. Although ATT incorporation reduces (tan δ)_max_ of neat PUAB, it shows minimal impact on Δ*T* and TA values, suggesting a moderate reduction in damping ability. Importantly, even with increasing ATT content, the 2 wt% ATT composite maintains a substantial (tan δ)_max_ of 0.93, demonstrating that the ATT-modified composites retain satisfactory damping performance.

### 3.7. Thermal Stability

**Figure 12:**
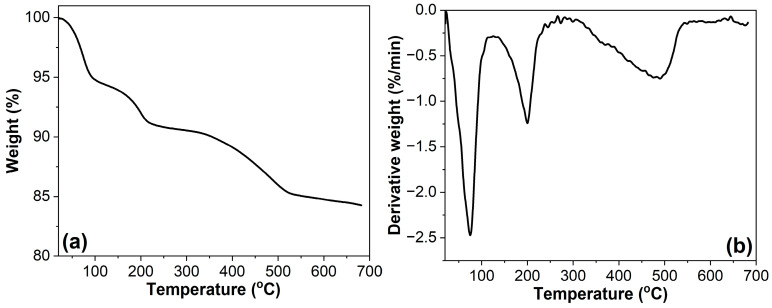
TGA (**a**) and DTG (**b**) curves of ATT.

The thermal decomposition behavior of pristine ATT was characterized by TGA and derivative thermogravimetry (DTG), as shown in Figure 12. For pristine ATT, the decomposition occurs in three distinct stages: the initial stage (20−110 °C, 5.1% weight loss), evolution of physically adsorbed water molecules; the intermediate stage (110−240 °C, 3.7% weight loss), loss of both zeolitic and hygroscopic water [67]; and the final stage (240−540 °C, 5.9% weight loss), dehydroxylation of structural hydroxyl groups.

**Figure 13:**
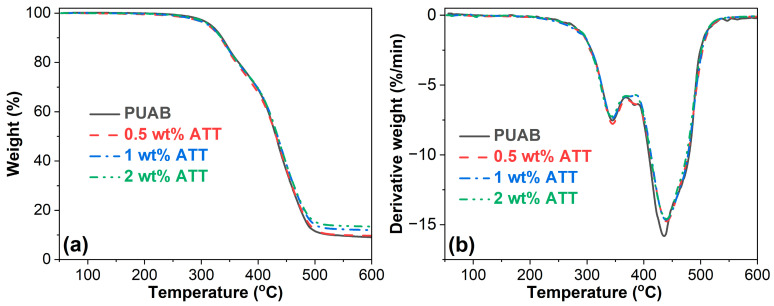
TGA (**a**) and DTG (**b**) curves of bio-based PUAB and PUAB/ATT composites.

The thermal decomposition of bio-based PUAB and its ATT composites differs markedly from pristine ATT, occurring in two primary stages (Figure 13): Stage I (250−360 °C, 26.1% weight loss), dissociation/reassociation of urethane linkages and thermal breakdown of bitumen’s low-molecular-weight saturates, aromatics, and resin fractions [68], and Stage II (360−540 °C, 61.4% weight loss), degradation of CO and residual polymer fragments from bio-based PUAB, concurrent with decomposition of bitumen’s asphaltene components [69].

Table 10 demonstrates the thermal characteristics of pristine ATT, bio-based PUAB, and PUAB/ATT composites obtained from TGA and DTG analysis. The initial decomposition temperature (*T*_i_), corresponding to 5% weight loss, measures 93.4 °C for pristine ATT, significantly lower than 317.4 °C. Incorporation of ATT moderately reduces the *T*_i_ of neat PUAB, though composite *T*_i_ values exhibit an increasing trend with attapulgite loading.

Pristine ATT shows two distinct decomposition peaks at 74.5 °C (*T*^1^_dmax_) and 119.4 °C (*T*^2^_dmax_), attributed to physically adsorbed water molecules, zeolitic water, and hygroscopic water. These peaks, along with the *T*^3^_dmax_ peak from hydroxyl groups, disappear in the composite DTG curves due to the polyaddition reaction between isocyanate groups (PU) and hydroxyl groups (ATT). While ATT addition negligibly affects the *T*^1^_dmax_ of neat PUAB, it causes a slight elevation in *T*^2^_dmax_. The relationship between ATT loading and decomposition temperatures (*T*^1^_dmax_ and *T*^2^_dmax_) follows a nonlinear pattern (Table 10).

The char residue at 600 °C increases from 9.1% (neat PUAB) to higher values with ATT incorporation, showing progressive enhancement at greater clay loading.

### 3.8. Mechanical Properties

**Figure 14:**
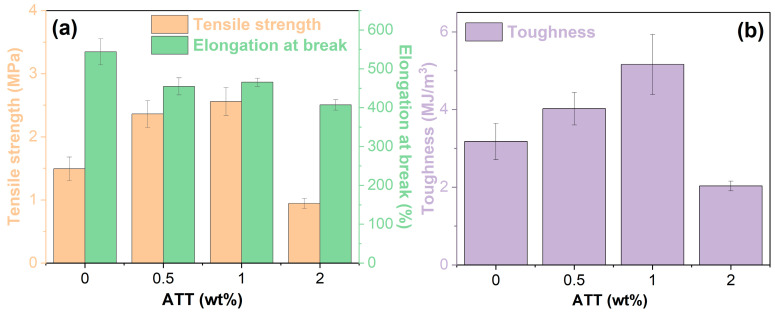
Tensile strength, elongation at break (**a**), and toughness (**b**) of bio-based PUAB and PUAB/ATT composites.

Figure 14 demonstrates the mechanical properties of bio-based PUAB and its ATT composites. As illustrated in Figure 14a, the tensile strength of neat PUAB (1.50 ± 0.19 MPa) increases by 58% and 71% with the addition of 0.5 and 1 wt% ATT, respectively. However, at 2 wt% ATT, the tensile strength decreases. Variations in the tensile strength of neat PUAB with ATT loading correlate directly with corresponding changes in crosslinking density, as quantified in Table 8. In contrast, ATT incorporation slightly reduces the elongation at break of neat PUAB, though all composites maintain high elongation (>400%). Mechanical properties of ATT composites with 0.5 and 1 wt% ATT satisfy the specification for thermosetting-polymer-modified asphalt (tensile strength ≥ 1.50 MPa and elongation at break ≥ 200%) [26].

Tensile toughness (τ) is defined as the area under the stress (σ) versus strain (ε) curve, calculated using Equation (5). This integral represents the energy per unit volume required to fracture the material.

(5) $$\tau =\int_{0}^{\varepsilon_{b}}\sigma d\varepsilon$$

where ε_b_ denotes the elongation at break. Similar to tensile strength, the incorporation of 0.5 and 1 wt% ATT improves the toughness of neat PUAB by 27% and 62%, respectively, whereas the 2 wt% loading reduces toughness. Notably, toughness improvement with 0.5 wt% and 1 wt% ATT loadings is limited compared to tensile strength enhancement, attributed to a slight reduction in elongation at break.

These significant improvements in the tensile strength and toughness are attributed to (1) the compatibilizing effect of ATT, which reduces interfacial tension between bitumen and polyurethane, leading to decreased bitumen particle size (Section 3.4), and (2) interaction between isocyanate groups in PU and hydroxyl groups in ATT (Section 3.1).However, ATT’s high surface energy promotes aggregation at higher loading (2 wt%), resulting in a significant reduction in tensile strength and toughness of bio-based PUAB [30].

## 4. Conclusions

Fibrous attapulgite was employed to enhance the mechanical properties of castor oil-derived bio-based polyurethane asphalt binder. The key findings are summarized below:

- FTIR spectroscopy confirms the reaction between isocyanate groups in PU and hydroxyl groups in ATT, which accelerates the conversion of the polyaddition reaction in PUAB.
- The addition of ATT reduces the rotational viscosity of bio-based PUAB during curing, thereby extending the allowable construction time. This viscosity reduction becomes more pronounced with increasing clay content.
- ATT decreases the bitumen particle size within the continuous polyurethane phase, with further reduction observed at higher clay loadings.
- While ATT incorporation slightly lowers both the glass transition temperature and damping properties of bio-based PUAB, it simultaneously enhances the material’s thermal stability.
- At loadings of 0.5 and 1 wt%, ATT significantly improves the tensile strength and toughness of bio-based PUAB, with only a marginal decrease in elongation at break.

Considering the combined effects on time-dependent rotational viscosity behavior, glass transition temperature, damping capability, thermal stability, and mechanical properties, 1 wt% ATT represents the optimum concentration for reinforcing bio-based PUAB.

## Figures and Tables

**Table 1:** Physicochemical characteristics of bitumen.

Properties	Standard	Value
Physical properties		
Penetration (25 °C, 0.1 mm)	ASTM D5-20 [37]	73.0
Ductility (10 °C, cm)	ASTM D113-17 [38]	15.8
Softening point (°C)	ASTM D36-06 [39]	48.2
Viscosity (60 °C, Pa·s)	ASTM D4402-06 [40]	173.0
Wax content (%)	ASTM D3344-90R21 [41]	1.83
Chemical components		
Saturates (%)	ASTM D4124-09R18 [42]	20.0
Aromatics (%)		31.5
Resins (%)		37.1
Asphaltenes (%)		6.8

**Table 2:** Chemical composition of attapulgite.

Oxides	SiO_2_	MgO	Al_2_O_3_	Fe_2_O_3_	K_2_O	TiO_2_	CaO	SO_3_	P_2_O_5_	MnO
Content (wt%)	57.72	9.75	9.12	4.70	1.07	0.86	0.34	0.15	0.08	0.05

**Table 3:** Heating and cooling stages for DSC measurements.

Stage	Process
First heating	After rapid cooling to −50 °C at maximum instrument capacity, heating to 100 °C at 20 °C/min
Thermal history erasure	3 min isothermal hold at 100 °C
Controlled cooling	20 °C/min ramp to −50 °C
Temperature equilibration	5 min isothermal hold at −50 °C
Second heating	20 °C/min ramp to 100 °C for glass transition temperature (*T*_g_) determination

**Table 4:** Vibration assignments of FTIR spectra for PUAB, PUAB/ATT composites, and their raw materials.

Peak Position (cm^−1^)	Assignment
3615	Stretching vibration of Al–Al–OH
3586	Stretching vibration of Al–Fe–OH
3550	Stretching vibration of bound water
3424	–OH group
3356	Stretching vibration of –NH
2965, 2924	Stretching –CH vibration of –CH_2_
2858	Symmetric stretching of –CH_2_
2247, 2264	–NCO group
1749	C=O group for ester
1712	Amide I: C=O stretching vibrations
1653	Bending vibration of bond water
1603, 866, 814	Vibration of aromatic rings
1508	–N–H in-plane bending
1460	Scissoring vibration of –CH_2_–
1452	Deforming vibrations of –CH–
1380	Umbrella vibration of –CH_3_
1366	Bending vibration of –CH_2_
1195	Stretching vibration of Si–O–Si
1166	C–O–C group
973	Deformation of M–OH
800	Si–O–Si symmetric stretching vibration of quartz
772	N–H out-of-plane bending
720	Sympathetic vibration of –(CH_2_)_n_–, *n* ≥ 4
645	Stretching vibration of H_2_O–Mg–H_2_O
581	Stretching vibration of AlO_6_ octahedron

**Table 5:** Conversions of bio-based PUAB and PUAB/ATT composites after 240 min curing.

ATT (wt%)	Conversion at 240 min (%)
0	92.0
0.5	92.4
1	92.1
2	94.6

**Table 6:** Time to reach 1 and 3 Pa·s for the rotational viscosity of bio-based PUAB and PUAB/ATT composites.

ATT (wt%)	Time to Reach 1 Pa·s (min)	Time to Reach 3 Pa·s (min)
0	60	90
0.5	63	96
1	68	101
2	69	105

**Table 7:** Average diameters and dispersity of bitumen domains in cured bio-based PUAB and PUAB/ATT composites.

ATT (wt%)	*D*_n_ (μm)	*D*_w_ (μm)	Ɖ
0	41.4 ± 2.0	50.7 ± 4.6	1.22
0.5	25.2 ± 0.7	36.0 ± 1.6	1.43
1	20.0 ± 0.6	25.7 ± 0.4	1.28
2	15.8 ± 0.2	21.6 ± 1.6	1.36

**Table 8:** *T*_g_s and crosslinking densities of bio-based PUAB and PUAB/ATT composites.

ATT (wt%)	υ_e_ (mol/m^3^)	*T*_g_ obtained from DSC (°C)	*T*_g_ obtained from DMA (°C)	
			E″	tan δ
0	24.8	−15.5	−5.4	10.7
0.5	34.5	−17.9	−7.2	9.4
1	25.4	−17.8	−6.7	9.4
2	20.4	−19.4	−6.8	9.3

**Table 9:** Damping parameters of bio-based PUAB and PUAB/ATT composites.

ATT (wt%)	(tan δ)_max_	Δ*T* (°C)	*TA* (K)
0	1.06	59.8 (−8.0~51.8)	41.8
0.5	1.06	59.1 (−8.7~50.4)	42.0
1	0.98	57.6 (−7.1~50.5)	38.4
2	0.93	59.1 (−7.9~51.2)	38.2

**Table 10:** TGA and DTG parameters for ATT, bio-based PUAB, and PUAB/ATT composites.

ATT (wt%)	*T*_i_ (°C)	*T*^1^_dmax_ (°C)	*T*^2^_dmax_ (°C)	*T*^3^_dmax_ (°C)	Char Residue at 600 °C (%)
0	317.4	344.0	435.6	-	9.1
0.5	312.0	344.9	439.9	-	9.7
1	313.8	344.4	439.1	-	12.0
2	316.1	342.9	440.0	-	13.4
100	93.4	74.5	119.4	489.0	84.8

## Data Availability

All data are available in the manuscript.
